# An LC-MS untargeted metabolomic comparison between three blood microsampling devices, whole blood, and plasma

**DOI:** 10.1007/s11306-026-02424-6

**Published:** 2026-05-29

**Authors:** Dennisse Avella, Soile Turunen, Glykeria Avgerinou, Anatoli Petridou, Vassilis Mougios, Iman Zarei, Seppo Auriola, Kati Hanhineva, Olli Kärkkäinen

**Affiliations:** 1 Afekta Technologies Ltd., Kuopio, Finland; 2 School of Pharmacy, University of Eastern Finland, Kuopio, Finland; 3 Department of Public Health and Clinical Nutrition, University of Eastern Finland, Kuopio, Finland; 4 School of Physical Education and Sport Science, Aristotle University of Thessaloniki, Thessaloniki, Greece; 5 Department of Life Technologies, Food Chemistry and Food Development Unit, University of Turku, Turku, Finland

**Keywords:** Blood microsampling (BµS), Dried blood spots (DBS), Volumetric absorptive microsampling (VAMS), Capitainer^®^, Liquid chromatography–mass spectrometry (LC-MS), Metabolomics

## Abstract

**Introduction:**

Blood microsampling (BµS) devices collect less than 100 µL of blood, offering a less invasive and more cost-effective alternative to venipuncture. However, its metabolomic comparability to conventional samples remains unclear, and standardized BµS metabolomic workflows are lacking.

**Objectives:**

This study evaluated the impact of using three BµS devices (Mitra^®^, Capitainer^®^, and Whatman^™^ 903) on the metabolomic interpretation of human biomonitoring samples. We compared them to conventional samples (plasma and whole blood) and evaluated the interplay of different analytical conditions.

**Methods:**

Venous blood from 10 adults (5 males, 5 females) was sampled onto the three devices. First, three agitation conditions (ultrasound, shaker, and homogenizer) were evaluated at three blood concentrations (1.5%, 5.5%, and 11%). The optimized method was then used to compare the metabolite profiles between BµS devices, whole blood, and plasma. Reverse-phase and hydrophilic-interaction chromatography, in positive and negative ionization modes, were combined for liquid chromatography–mass spectrometry (LC-MS) analysis.

**Results:**

All agitation conditions and concentrations proved suitable for BµS untargeted metabolomics. Combining different analytical modes and fragmentation ranges proved helpful for maximizing metabolite coverage. BµS-derived metabolite profiles aligned more closely with whole blood than plasma. Some metabolites were more characteristic of a sample type, whereas others were common across sample types. All sample types enabled sex-based differentiation, with metabolites such as amino acids, lipids, and acylcarnitines driving the separation.

**Conclusions:**

These findings enhance our understanding of BµS metabolite coverage and highlight its potential in human biomonitoring. The choice of device depends on the application and the metabolites of interest, offering flexibility for clinical use and research.

**Supplementary Information:**

The online version contains supplementary material available at https://doi.org/10.1007/s11306-026-02424-6.

## Introduction

Minimally invasive and remote sampling is more convenient in human health biomonitoring, as people report less pain sensation in comparison to conventional blood collection (Solheim et al., 2021). Conventional sampling requires several milliliters of samples and specialized sampling personnel, among other limitations (Couacault et al. ,^2024^). In contrast, blood microsampling (BµS) techniques, such as the conventional dried blood spots (DBS), typically collected on Whatman^™^ 903 cards since the 1960s, and newer quantitative approaches, such as volumetric DBS (e.g., Capitainer^®^) and volumetric absorptive microsampling (VAMS, e.g., Mitra^®^), require only a few microliters of blood that can be self-collected, facilitating decentralized sampling and analysis, and more important, reducing the discomfort in vulnerable groups of people (Uytfanghe et al., 2021). However, concerns remain on how well the BµS represent conventional venous samples, particularly in untargeted metabolomics applications (Hernandes et al., 2017).

Most of the previous literature has focused on metabolomics and lipidomics studies on Whatman™ samples, as this sampling device has been integrated into clinical research for several years (Guo et al., 2023; Hernandes et al., 2025). Similarly, the use of Mitra^®^ devices has gained attention mainly due to their quantitative and practical sampling benefits (De Sá et al., 2023). Most recently, other types of volumetric DBS, like Capitainer^®^ and Tasso-M20, or plasma separation cards, such as Telimmune, have started to attract interest (Londoño-Osorio et al., 2025; Bossi et al., 2025; Cendali et al., 2023; Thaitumu et al., 2025). However, most of the studies have focused on targeted applications. Even within untargeted metabolomics studies, the extent to which BµS represent the metabolite profile of whole blood remains unclear (Chiu et al., 2024; Shi et al., 2024; Volani et al., 2023; Couacault et al., 2024). The primary concern is that the limited sample volume and matrix effects introduced by the BµS devices could dampen the detection of biological differences in metabolomics studies.

Therefore, we aimed to evaluate the impact of using BµS devices (Mitra®, Capitainer®, and Whatman™) on the blood metabolite profile and related biological information, from an untargeted metabolomics perspective. We investigated how well metabolite-level information from conventional matrices is preserved when using BµS devices, and whether there are significant differences that could affect biological interpretations.

## Methods

This study is part of the HUMAN (Harmonising and Unifying Blood Metabolomic Analysis Networks) Doctoral Network (human-dn.eu), funded by the European Union. In this part of the project, five labs received five sample types (Mitra^®^, Capitainer^®^, Whatman^™^, whole blood, and plasma) prepared from venous blood, a standardized homogeneous source, to gain a broader understanding of the samples’ comparability through different approaches. In our lab, we specifically aimed to evaluate the impact of using BµS devices on the blood metabolite profile and the related biological information, from an untargeted metabolomics perspective. A comparison with the related consortium studies is presented in the Supplementary Material 1 Table 8.

Briefly, our untargeted metabolomics approach included (1) whole blood to compare the profile that the BµS devices could capture from the red blood cells, (2) plasma, as it remains the “gold standard” for comparison with other clinical metabolomic studies (Couacault et al., 2024), and (3) the evaluation of the interplay of different analytical factors: three agitation conditions, three sample concentrations, the complementarity of four analytical liquid chromatography–mass spectrometry (LC-MS) modes (reverse-phase (RP) and hydrophilic-interaction chromatography (HILIC), in positive (+) and negative (–) ionization), and the impact of using several tandem mass spectrometry (MS/MS) fragmentation ranges, and device blanks.

### Materials

All solvents were high-performance liquid chromatography (HPLC) grade: Methanol (MeOH, CHROMASOLV LC–MS Ultra) was obtained from Riedel-de Haën, Honeywell (Seelze, Germany); acetonitrile (ACN, HiPerSolv CHROMANORM) from VWR Chemicals (Fontenay-sous-Bois, France); and formic acid (Optima LC/MS) from Fisher Chemical (Geel, Belgium). Ultrapure water was generated using a Milli-Q system.

The BµS devices were Mitra^®^ VAMS (Neoteryx), Capitainer^®^ B (Capitainer), and Whatman™ 903 cards (Sigma-Aldrich). Each Mitra^®^ contained four 20 µL tips, Capitainer^®^ cards had two 10 µL collection spots, and Whatman™ cards included five collection spots. Additional consumables included Microcentrifuge tubes, 1-mL syringes, 0.2-µm polytetrafluoroethylene (PTFE) syringe filters, and LC-MS-compatible vials and caps.

### Sample collection

Venous blood was collected from 10 healthy adult volunteers (5 males, 5 females) at the Aristotle University of Thessaloniki with informed consent and ethical approval. A pooled 11th sample was prepared by combining equal aliquots from the 10 individual samples. All participants fasted overnight before venous blood collection. Demographic characteristics are presented in Supplementary Material 1. Capillary blood was not used as we needed a homogeneous starting point to be distributed among five labs; therefore, this study did not yet evaluate the direct applicability to capillary samples.

The venous blood samples were divided into: whole blood, plasma, and BµS devices: Mitra^®^, Capitainer^®^, and Whatman™. Plasma was obtained by centrifugation; Mitra^®^ tips were dipped into whole blood; Capitainer cards were prepared by pipetting 10 µL onto each collection spot; and Whatman cards were prepared by pipetting 20 µL onto each spot. Whole blood and plasma were immediately stored at − 80 °C, and the BµS devices were stored at the same temperature after 3 h drying at room temperature. After seven days under these conditions, they were shipped on dry ice for 3 days and re-stored at − 80 °C before analysis.

### Optimization of sample preparation

Agitation conditions were first tested on Whatman™ triplicate samples using three sample volumes: ≈ 3 µL (3-mm punch), ≈ 11 µL (6-mm punch), and ≈ 22 µL (two 6-mm punches). The approximate sample concentrations were 1.5% (µL blood/µL extraction mixture), 5.5%, and 11%, respectively. Whatman™ blanks were prepared at the same punch sizes. The extraction protocol included 40 µL of H_2_O followed by 160 µL of MeOH/ACN (1:1 (v/v)), vortexing for 10 s after each volume addition. Three agitation conditions were tested: ultrasound, shaker, and homogenizer: (1) 2 min ultrasound followed by 15 min on ice, (2) 15 min shaking (520 rpm), and (3) 2 min homogenization (4 °C at 4 m/s on an OMNI Bead Mill Homogenizer (no beads were used)) followed by 15 min on ice. Then, the samples were centrifuged (10 min at 17,000×g, 4 °C), and supernatants were filtered (0.2 μm PTFE) before LC-MS analysis. Quality control (QC) samples were prepared by pooling aliquots from all samples. The sample concentration and agitation conditions were varied simultaneously to assess their combined effects.

For the optimization, we chose Whatman™ samples as they are the most studied BµS device (Couacault et al., 2024). Additionally, it facilitated the evaluation of different sample concentrations by varying the punch size. On the other hand, the ultrasound, shaker, and homogenizer, and the composition of the extraction mixture were chosen after realizing that several authors have used one of these agitation methods and have already studied, optimized, and preferred this composition (Couacault et al., 2024; Ward et al., 2021). Supplementary Material 1 Table 1 summarizes the conditions used by other authors, and Table 2 summarizes those we evaluated.

### Extraction across devices

The optimized sample preparation protocol was applied to whole blood, plasma, Mitra^®^, Capitainer^®^, and Whatman™ triplicate samples, prepared from the pooled sample. A single, standardized sample volume of 20 µL was used for all samples. According to the manufacturers’ intended specifications, one Mitra tip of 20 µL and two Capitainer spots of 10 µL were used. The collected samples were placed into Microcentrifuge tubes. For all sample types, 80 µL of H_2_O and 320 µL of MeOH/ACN (1:1 (v/v)) were added, vortexing for 10 s after each volume addition, shaking for 15 min at 520 rpm, centrifugation for 10 min at 17,000×g, 4 °C, and filtration (0.2 μm PTFE). The sample preparation and analysis order were randomized using the Wranglr tool (antonvsdata.shinyapps.io/wranglr). Pooled QC samples and blanks were included.

After demonstrating that the optimized extraction method was suitable for the five sample types, it was applied to the 5 male and 5 female samples, as well as the pooled sample, for all five sample types. No replicates were included. One individual’s whole blood sample (E05B) was not received. The pooled plasma and whole blood were mixed from equal volumes of individual samples.

### LC-MS analysis

Untargeted metabolomics analysis was performed using RP and HILIC, coupled with a quadrupole-time-of-flight mass spectrometer (Q-TOF), in both + and – ionization modes, with full-scan MS and MS/MS data acquired. Full details are available in Supplementary Material 1 and in a previous publication (Klåvus et al., 2020).

### Quality control

Absolute quantification and analytical method validation were not contemplated in this study, as the untargeted investigation comprises thousands of diverse metabolites; therefore, internal standards (ISs) were not included. However, the quality of the analysis was monitored by injecting a system suitability test (SST) standard solution at the beginning to verify retention times and abundances. More information on the composition of the SST solution is in Supplementary Material 2. Additionally, QC samples were injected throughout the analysis to stabilize instrument performance and to monitor and ensure the high quality of the acquired and processed data. The QC preparation, injection frequency, and metrics are provided in Supplementary Material 3 and 4.

### Data analysis

The Mass Hunter Qualitative (Version B.07.00, Agilent Technologies) software was used to verify the analytical performance on the SST mixture. Raw data were exported to MS-DIAL (version 4.9.221218), where peak detection and alignment were performed (Tsugawa et al., 2020). Detection parameters included a minimum peak height of 5000 counts and a width of 8 scans. Mass tolerances were 0.01 Da (precursor ions mass spectrometry (MS1) and 0.05 Da (MS/MS); retention time (RT) tolerance was 0.1 min. Features not exceeding a threshold of 5 compared to the blank solutions were neglected.

The aligned data was pre-processed with the R-package notame (Klåvus et al., 2020) in RStudio (2023.12.1 version 3). Features were log-transformed, drift-corrected using cubic splines, and filtered based on reproducibility and detection thresholds. Features with > 70% presence in QCs, > 50% in at least one group, relative standard deviation (RSD) < 20%, and D-ratio < 40% were retained (considered high-quality features) and imputed using random forest for missing values (Klåvus et al., 2020).

Metabolite identification was performed using both in-house and public databases (Human Metabolome Database, n.d.; LIPID MAPS, n.d.; MassBank of North America, n.d.). MS-DIAL putative reference matched identifications, focusing on chromatogram quality, retention time, mass-to-charge ratio (*m/z*) values of molecular ions, and MS/MS fragmentation spectra, were prioritized, manually inspected, and ranked at identification level 1 to level 4 (Sumner et al., 2007). We excluded redundant ions representing the same metabolites.

To assess significant metabolomic differences between devices and their ability to detect biological differences, statistical analyses were performed using Welch’s t-test and Cohen’s d (*p* ≤ 0.05, |d| ≥ 0.8). Cohen’s calculations used each sample type’s individual standard deviations.

Visualizations included heatmaps, principal component analysis (PCA) plots, Venn diagrams, volcano plots, and QC boxplots, created using Excel, RStudio, MetaboAnalyst 6.0 (metaboanalyst.ca), and VolcaNoseR (Goedhart & Luijsterburg, 2020).

## Results

### Optimization of sample preparation

Optimizing sample preparation conditions was an essential first step toward a better understanding of BµS in untargeted metabolomics analysis. For this purpose, we initially focused on DBS using Whatman^™^ samples to evaluate metabolite extraction and distinguish analytical signals. We categorized features as blood-derived, paper-associated, and background-related. Blood-derived features were those with a high response in samples but an insignificant response in blank solutions; paper-associated features presented a higher response in blank solutions containing paper, and their response was proportional to the amount of paper; and background-related features were those with a constant response across all samples and blanks or with a higher response in the background blank solution. The intensity of some features scaled proportionally to the concentration, while others, were less proportional, indicating a volume-dependent effect on recovery.

Then, we compared the ultrasound, homogenizer, and shaker across low (1.5%), medium (5.5%), and high (11%) blood concentrations (µL sampled blood/µL extraction mixture). Overall, the three agitation conditions demonstrated to be viable for the extraction purposes, as there was a non-significant difference between them, regarding the blood metabolites’ detection or abundance. We selected shaking as the preferred agitation method for subsequent analyses, as ultrasound may promote molecular degradation due to heat generation, and the homogenizer requires dry ice to maintain a controlled temperature.

Among the four analytical modes tested, RP+ detected the most features, while HILIC- detected the fewest. This confirmed that the extraction conditions could retrieve meaningful metabolite information. Among the blood-derived compounds, we identified lipids and metabolites like phosphatidylcholine 34:1 (16:0_18:1), hippuric acid, and pyrocatechol sulfate. Supplementary Material 5 Table 1 provides the putative identifications.

Most blood-related features increased proportionally with sample concentration. However, at higher concentrations, signal suppression was noted in five metabolites, including phosphatidylcholines in RP + and inosine 5’-monophosphate in HILIC+. Phosphatidylcholines’ responses were more proportional in RP-. Overall, low-to-medium sample concentrations were preferred to minimize signal suppression.

Background signals presented high intensity in the method-workflow blank. However, background amides remained consistent across blanks and samples. Additionally, the signal of some RP-blank features also increased in proportion to blood concentration, necessitating careful interpretation. Finally, the paper-associated signals, similarly to blood-related features, were not affected by the agitation conditions.

### Extraction across devices

We tested the consistency of the optimized shaker-based extraction across whole blood, plasma, Mitra^®^, Capitainer^®^, and Whatman^™^ samples, at 5% concentration.

**Fig. 1:**
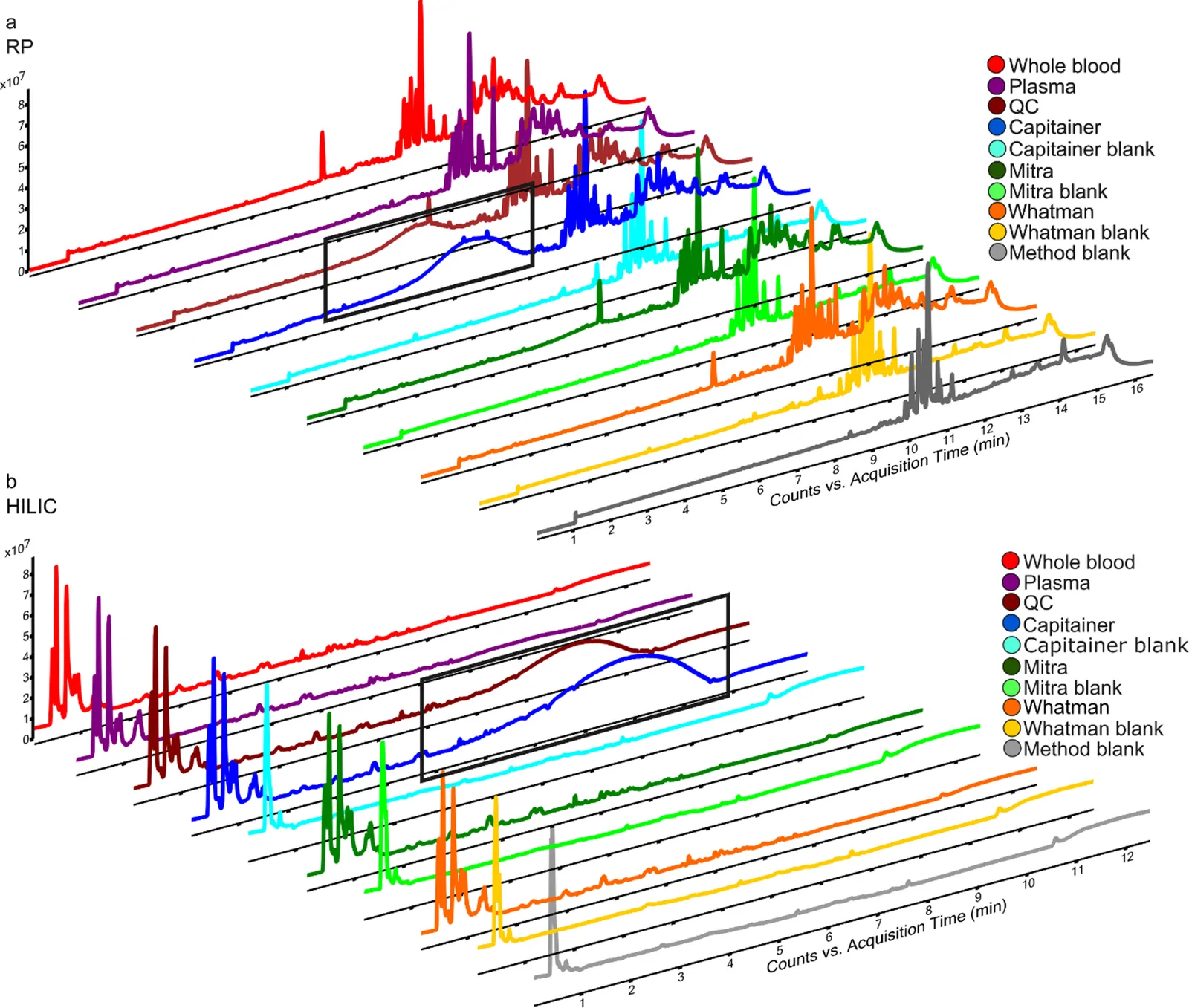
Comparison of the total ion chromatograms of samples and blanks in two of the four analytical modes (**a**) reverse phase chromatography with positive ionization (RP+) and (**b**) hydrophilic-interaction chromatography with positive ionization (HILIC+) modes. A repeating *m/z* unit (44 Da) in Capitainer^®^ samples suggests polyethylene glycol interference from surface additives during blood interaction. (highlighted in the black squares). A similar behaviour was observed in the negative modes

The total ion chromatograms (TIC) revealed, in all modes, a recurring interfering signal in Capitainer^®^ and QC samples (containing Capitainer® aliquots) (Fig. 1), attributed to polyethyleneglycol (PEG) with a 44 Da repeating unit. This signal was not present in Capitainer^®^ blanks but appeared in Capitainer-blood-containing samples.

### Combination of LC-MS modes and fragmentation ranges

Following the optimization of the sample preparation conditions, we explored the contribution of combining analytical modes (RP+, RP-, HILIC+, and HILIC-) in BµS untargeted metabolomics. We initially detected 16.553 features; 10.940 passed blank filtering. Among them, 6.003 were found in RP+, 1.555 in RP-, 2.740 in HILIC+, and 642 in HILIC-. The quality data differentiated 4,624 as high-quality features.

MS/MS annotation yielded 151 unique metabolites. RP+ detected the highest number of features and identified metabolites, while HILIC- had the fewest (Supplementary Material 5 Table 2). All metabolites were technically detected across all sample types due to MS-DIAL gap-filling, but those with a fold change below 2, relative to method or device blanks, were considered undetected to avoid inclusion of background noise signals. Final counts of identified metabolites were: 141 (whole blood), 134 (plasma), 146 (Mitra^®^), 145 (Capitainer^®^), and 148 (Whatman™) (Supplementary Material 5 Table 3). Supplementary Material 5 tables compare data across sample types, statistical information, and metabolites that were excluded, common, and unique by sample type, analytical mode, and volunteers’ sex.

Six metabolites were detected in all samples except plasma: 3-carboxaldehyde, 7-hydroxycholesterol, 7-ketocholesterol, tetracosanoic acid, lysophosphoethanolamine 18:0, and histamine. Alpha-tocopherol was found only in whole blood and plasma. Five metabolites were detected only in BµS, including 4-hydroxybenzaldehyde, phosphatidylcholines, fatty acids, and sterols, indicating complementary coverage between BµS and conventional samples (Supplementary Material 5 Table 4).

**Fig. 2:**
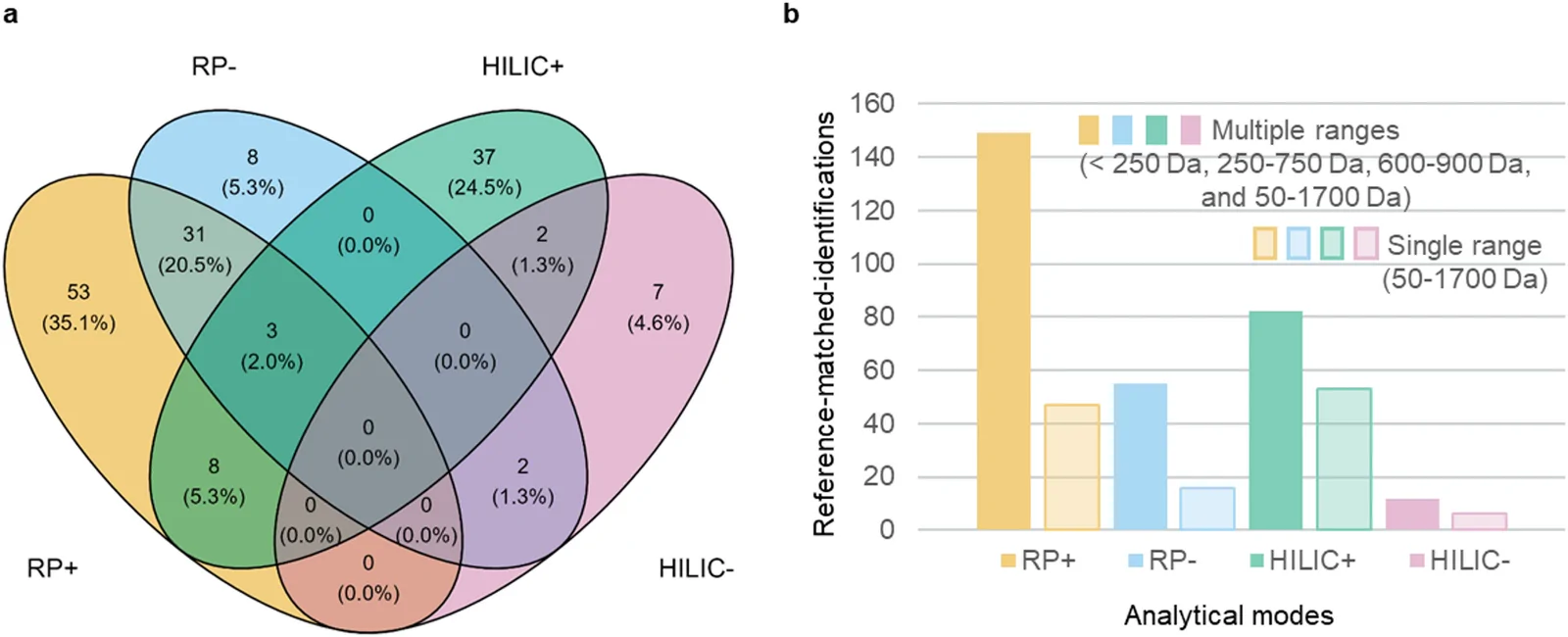
**a** Venn diagram representing the number of identified metabolites common and unique across analytical modes, and (**b**) bar diagram showing how the number of identified metabolites increased in all the analytical modes when using multiple fragmentation ranges. The evaluated mass ranges were a single range (50-1700 Da) vs. multiple ranges (< 250 Da, 250–750 Da, 600–900 Da, and 50-1700 Da)

To assess the contribution of the 4 modes, we evaluated how many of the 151 metabolites were common or unique across them (Fig. 2a). RP+ uniquely identified 53 metabolites, mainly phospholipids and methylxanthines. HILIC+ uniquely detected 37 metabolites, mainly amino acids and nitrogenous compounds. RP- and HILIC- identified fewer unique metabolites, including sulfates and organic acids (Supplementary Material 5 Table 5). Overlaps between modes helped verify lipid identifications, while better ionization of most metabolites was observed in positive mode.

Additionally, we assessed the impact of using multiple narrower MS/MS fragmentation ranges (< 250 Da, 250–750 Da, 600–900 Da, and 50-1700 Da) compared with a single broad range (50-1700 Da) in auto MS/MS during data acquisition (Fig. 2b). In all analytical modes, using multiple narrow fragmentation ranges improved metabolite identification.

### Device and sex based metabolic differentiation

**Fig. 3:**
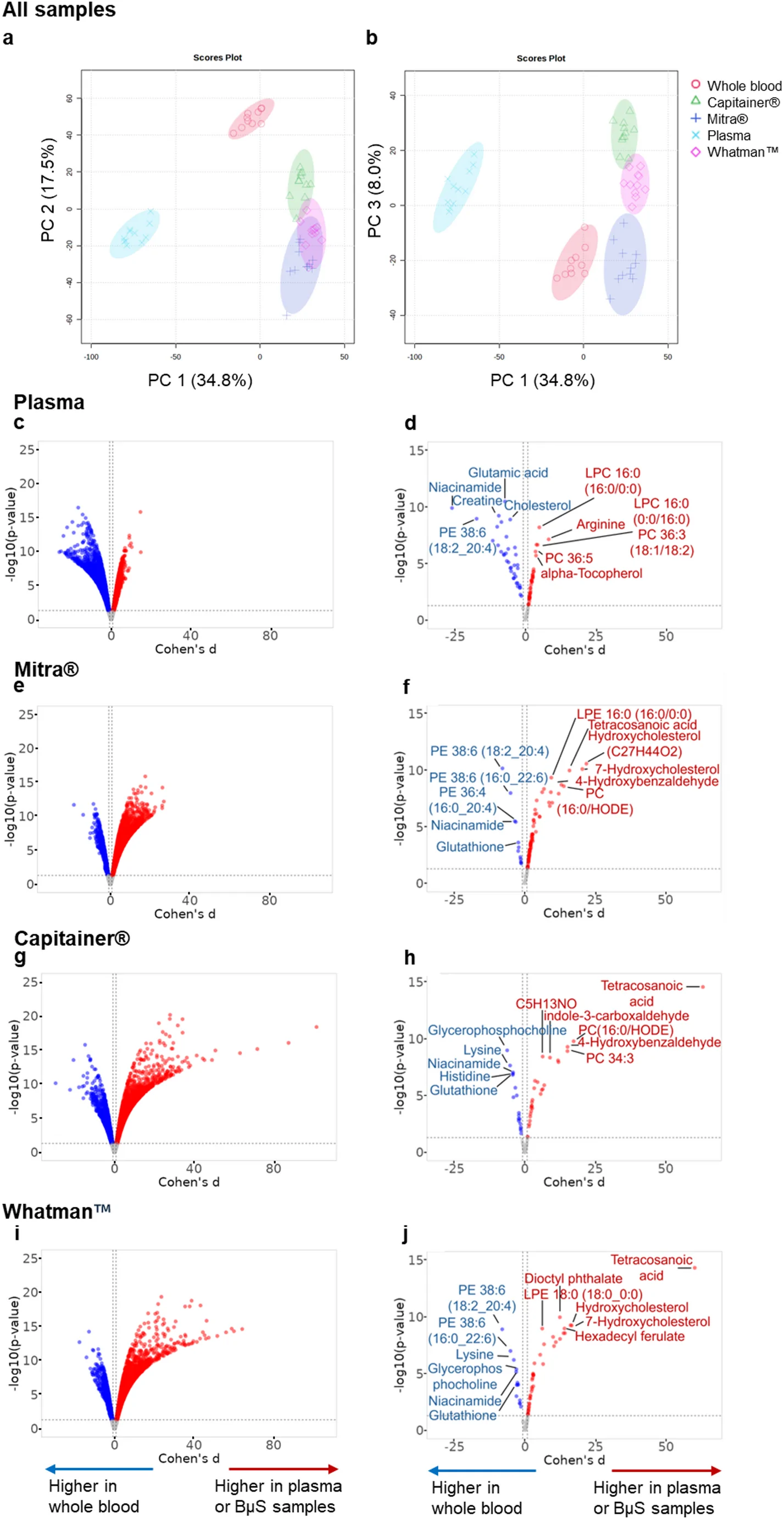
Differences in metabolite coverage between whole blood and other sample types. Principal component analysis (PCA) score plots from analysis of the high-quality features of all samples showing (**a**) PC1-PC2, (**b**) PC1-PC3, and (**c** to **j**) Volcano plots comparing plasma and BµS with whole blood, all detected features are on the left; annotated metabolites are on the right. Red indicates features/metabolites higher in plasma or BµS samples; blue indicates higher levels in whole blood

To further explore the metabolite coverage differences between BµS and conventional samples, we performed PCA (4.624 high-quality features). PC1 separated plasma from red blood cell-containing samples (Fig. 3). PC2 (Fig. 3a) and PC3 (Fig. 3b) differentiated whole blood from BµS and separated BµS devices, respectively. In PC2, whole blood samples were clustered more closely with Capitainer^®^ samples (Fig. 3a), while in PC3, whole blood samples were closer to Mitra^®^ samples (Fig. 3b). Among devices, Mitra^®^ (VAMS polymer-tips) was differentiated from Capitainer^®^ and Whatman™ (paper-based DBS). In both planes, Mitra^®^ samples were closer to Whatman™ than to Capitainer^®^. Despite the PEG interference, Capitainer^®^ samples closely matched whole blood molecular feature abundances.

We focused our investigation on the metabolic comparison with whole blood. The volcano plots in Fig. 3 show the features and annotated metabolites varying between the BµS samples, plasma, and whole blood. Mitra^®^ had the highest number of features significantly enriched when compared with whole blood, followed by Whatman^™^, Capitainer^®^, and plasma. Among annotated metabolites, 102 were higher in BµS or plasma, and 47 in whole blood. The lists of significant metabolites per sample type are presented in Supplementary Material 5 Table 6 (higher in BµS or plasma than in whole blood) and Table 7 (higher in whole blood than in BµS or plasma).

Mitra^®^ showed the greatest number of unique metabolites, with 29 metabolites significantly higher in Mitra^®^ than in whole blood, mostly amino acids and acylcarnitines. Plasma presented 11 unique metabolites, mainly phosphatidylcholines. Capitainer^®^ and Whatman^™^ enriched fewer unique metabolites, 1 (fumaric acid) and 3 (dioctyl phthalate, hexadecyl ferulate, and phosphatidylcholine 34:2), respectively. Tetracosanoic acid was elevated in all BµS types but was also present in Capitainer^®^ and Whatman^™^ blanks, hindering the interpretation of its biological relevance (Supplementary Material 5 Table 6).

In contrast, 22 unique metabolites were higher in whole blood compared with plasma, mainly amino acids and carnitines. 6 unique metabolites were elevated in whole blood compared with Capitainer^®^, while 1 unique metabolite compared with Whatman^™^, and no unique metabolites were higher in whole blood than in Mitra^®^. 5 metabolites were higher in whole blood than in all other samples, including niacinamide, glutathione, and some phosphatidylethanolamines (Supplementary Material 5 Table 7).

**Fig. 4:**
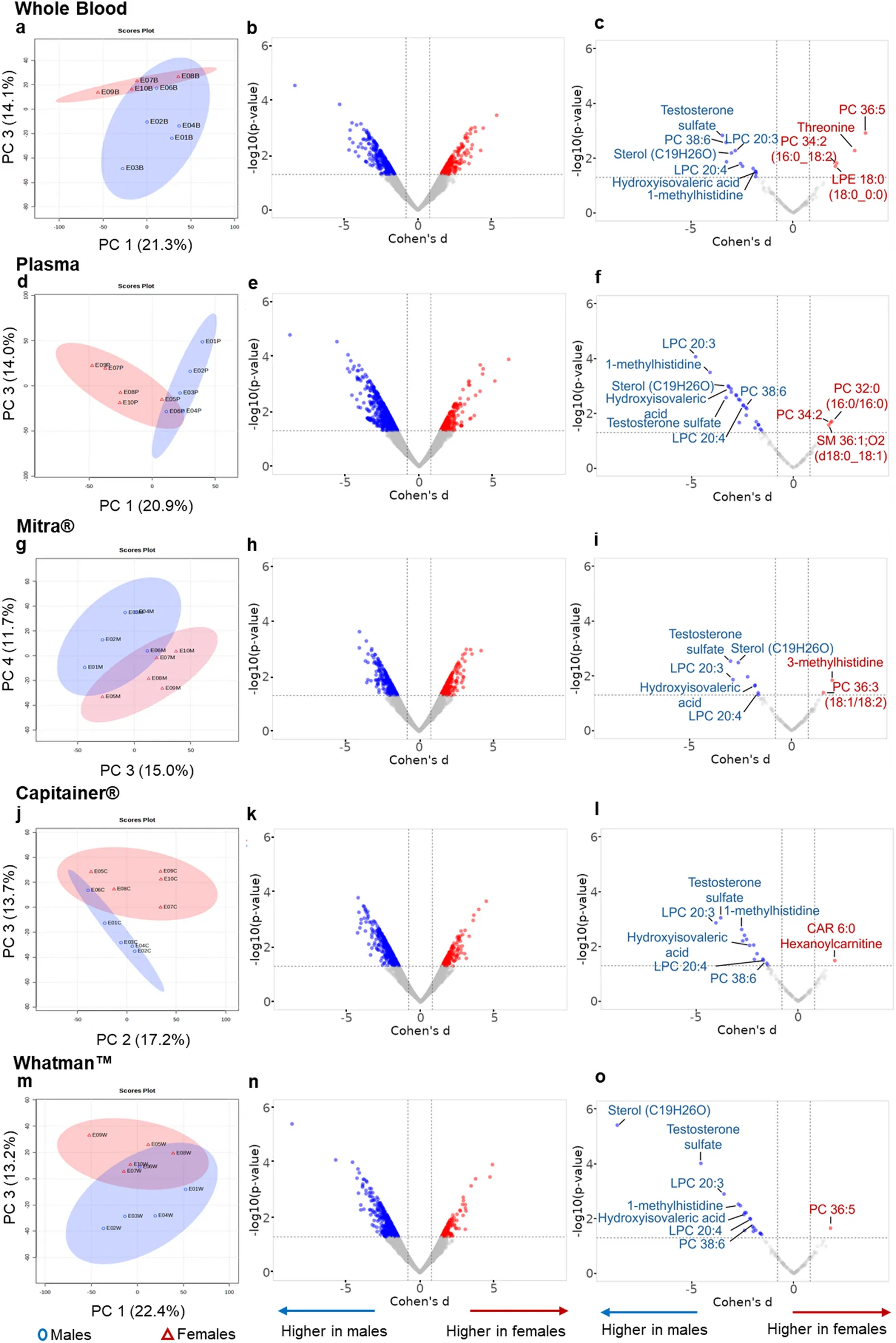
Sex-based differentiation to assess the capability of the sample types to reflect biological differences. Principal component analysis (PCA) score plots and volcano plots comparing male and female samples by sample type using the high-quality features. (**a**, **d**, **g**, **j**, **m**) PCA plots are on the left; (**b**, **e**, **h**, **k**, **n**) volcano plots of all detected features are in the center; (**c**, **f**, **i**, **l**, **o**) volcano plots of annotated metabolites are on the right. Red indicates higher levels in females and blue higher levels in males

Sex-based differentiation assessed the capability of BµS to reflect biological differences. The score plots on Fig. 4 showed differentiation between samples from males and females, with every sample type; however, cluster separation was most evident in plasma, Mitra^®^, and Capitainer^®^ (Fig. 4d, g, j), with only the sample of the sixth volunteer overlapping between groups (whole blood missed one female sample that was not received before the analysis). The volcano plots showed that significant sex-related metabolites were higher in males (32) than in females (10, Fig. 4). Female-associated metabolites were device-specific, including PCs and sphingomyelins, while male-associated metabolites were more common across sample types. Lists of significant sex-differentiated metabolites are presented in Supplementary Material 5 Table 8 (higher in females) and Table 9 (higher in males).

Sex differentiation was mainly driven by metabolites higher in males; none were unique to whole blood. Of the 12 significant metabolites identified in whole blood, at least 10 were also significant in plasma, Capitainer^®^, and Whatman^™^, while 6 were significant in Mitra^®^. Plasma exhibited the highest number of unique metabolites, with 6 metabolites; still, it shared 14 significant metabolites with one or more BµS. Fewer metabolites were unique to the BµS devices. Overall, 11 metabolites were shared between two or more sample types, and 4 metabolites were commonly significant across the five sample types: testosterone sulphate, hydroxyisovaleric acid, lysophosphatidylcholine 20:3, and lysophosphatidylcholine 20:4 (Supplementary Material 5 Table 9).

## Discussion

This study evaluated the biological equivalence of untargeted metabolite profiles obtained from three BµS devices (Mitra^®^, Capitainer^®^, and Whatman^™^) compared to conventional matrices (plasma and whole blood). By optimizing sample preparation conditions, combining four LC-MS modes, and comparing statistical and metabolomic differences between samples, we have demonstrated that the BµS devices’ matrix does not affect the biological interpretation compared with conventional samples. The similarity of BµS metabolite profiles, especially to those of whole blood, and their ability to detect biological differences make them a suitable option for human untargeted metabolomic studies.

We optimized an extraction protocol suitable for the five sample types. We selected 15-minute room-temperature shaking at 5% concentration for its convenience. Various BµS agitation conditions have been reported (Couacault et al., 2024). Several of them include the extraction mixture we used and shaking as the preferred agitation technique (Alodaib et al., 2023; Guo et al., 2023; Sebaa et al., 2023). Previous studies have compared the impact of different extraction solvents (Bossi et al., 2025; Thaitumu et al., 2025). However, to our knowledge, our study is the first to show that different agitation conditions can lead to similar results.

Furthermore, combining RP and HILIC under positive and negative ionization modes enhanced metabolite coverage. Results from other authors support the benefits of combining RP and HILIC chromatography (Assress et al., 2025; Couacault & Witting, 2026). Metabolite classes uniquely detected, such as amino acids in HILIC+, and common across modes, such as lipids in RP+, and RP-, highlighted the modes’ complementarity. Additionally, the use of multiple MS/MS fragmentation ranges further improved metabolite identification.

All BµS devices successfully reflected key features of whole blood, although their performance varied by metabolite class. Mitra^®^ excelled in detecting amino acids and acylcarnitines. Capitainer^®^ and Whatman^™^ offered broader comparability with whole blood, while plasma remained preferable for certain lipids, such as phosphatidylcholines. These findings align with those of Thaitumu et al. (2025), who reported similar trends in compound detection. Higher abundances in Mitra^®^ samples could be due to better desorption of polar metabolites from the polymeric tips compared to cellulose-based devices, or to overloading if the tips are immersed in blood. Consistent with Londoño-Osorio et al. (2025), phosphatidylcholines, more abundant in plasma than in red blood cells, were more readily detected in plasma samples.

Importantly, similarly to whole blood and plasma, BµS devices enabled sex-based metabolic differentiation, with male-associated biomarkers well preserved. This supports prior findings that microsamples can reliably capture biological variation (Petrick et al., 2024; Chiu et al., 2024; Cendali et al., 2023). Previous studies support some of the main metabolic differences we found, suggesting higher concentrations of some sphingomyelins and phosphatidylcholines in females and higher concentrations of some lysophosphatidylcholines, amino acids, and acylcarnitines in males (Mittelstrass et al., 2011). Some examples of male upregulated amino acids and carnitines, common to our study, include valine, leucine, isoleucine, tryptophan, proline, propionylcarnitine (CAR 3:0), and isovalerylcarnitine (CAR 5:0) (Costanzo et al., 2022; Mittelstrass et al., 2011).

Previous sex-differentiation studies have been conducted on samples such as plasma, serum, or urine (Costanzo et al., 2022), not with whole blood. Similarly, while earlier studies often compared BµS to plasma, our inclusion of whole blood provides additional insight, showing that BµS preserves cellular metabolite signatures that plasma may miss (Volani et al., 2023).

Among our main strengths, our study is a comprehensive untargeted metabolomic comparison of three different BµS devices: Mitra^®^, Capitainer^®^, and Whatman^™^ with whole blood, expanding upon earlier work that has focused on plasma. Our approach is distinctive in its simplicity, avoiding evaporation or solvent exchange, and performing consistently across all studied matrices. And we include the evaluation of the collective role of different analytical variables, such as sample concentration, agitation conditions, and LC-MS parameters.

Among our study design limitations, the small cohort size limited the statistical power. We especially acknowledge that we did not receive the whole blood sample from the fifth volunteer. Despite the small cohort, metabolic distinctions between sexes were evident across all analyzed sample types. The menstrual status was not considered, as it remained constant across the sample types that were the focus of the comparison.

Further limitations include the use of venous blood instead of capillary blood, which may behave differently. We required a large volume per participant; therefore, pricking for capillary blood would not have been appropriate. In future studies, BµS metabolomes need to be evaluated using capillary blood. However, it should be noted that previous studies have shown good metabolomic overlap between venous and capillary samples using Mitra^®^ (Volani et al., 2023), and minimal metabolome differences across blood collection methods (Zubkowski et al., 2025), supporting the comparability between venous and capillary blood.

We also want to highlight that all our samples are always filtered to remove any possible particulates from the matrices to protect the instruments and to increase sample consistency and data quality. The five sample types and blanks were filtered under the same conditions; therefore, we expect a similar systematic effect of the filtration step in all the samples.

Regarding the devices, blood absorption on them and the practicality of collecting samples were limiting factors. When we collected the samples, Mitra^®^ proved more user-friendly; however, immersing the tip into blood could overfill it. Capitainer^®^, though accurate in volume, exhibited slower diffusion of cold venous blood and often left sample residue on the device. Whatman^™^ required punching tools and is known to be affected by hematocrit-related diffusion issues. These handling differences could affect the amount of sampled blood, reproducibility, and the risk of contamination.

Finally, the analysis of device blanks proved essential. For example, we detected PEG in Capitainer^®^ samples with blood, suggesting that PEG on the Capitainer^®^ surface was released upon blood interaction and not by the solvent mixture extraction (Jafar Mazumder et al., 2019). Also, tetracosanoic acid was detected in samples and both Capitainer^®^ and Whatman^™^ blanks, reinforcing the need for background correction in untargeted analyses.

## Conclusions

This study highlights the potential of Mitra^®^, Capitainer^®^, and Whatman^™^ BµS devices for untargeted metabolomics, offering metabolite coverage comparable to whole blood and plasma, with the added benefits of ease of use and suitability for remote sampling. While plasma remains optimal for some lipid analyses, BµS devices captured broader metabolite signatures from whole blood and allowed biological differentiation.

Ultimately, the metabolite class of interest should guide the choice of microsampling device, followed by practical and analytical considerations. Overall, with the metabolomic similarities to conventional samples and analytical benefits presented in this study, BµS devices are recognized for offering minimally invasive sampling, suitability for remote collection, and enhanced stability. These advantages support broader implementation of BµS devices in clinical research and human biomonitoring.

## Supplementary Information

Below is the link to the electronic supplementary material.

Supplementary Material 3: Quality Control (QC) solution information and metrics.

Supplementary Material 1: Methods

Supplementary Material 7: Additional results figures.

Supplementary Material 2: System Suitability Test Standard Solution Mixture Information.

Supplementary Material 4: QCs’ metabolites abundances and RSD calculations.

Supplementary Material 6: Extraction replicates' RSD calculations.

Supplementary Material 5: Results.

## Data Availability

Data and analysis code will be available to any interested researcher upon request from the corresponding author. The metabolomic data and metadata will be available on the MetaboLights public database [ebi.ac.uk/metabolights], study identifier [ **MTBLS10740** ]. The general code of notame is available via [github.com/hanhineva-lab/notame].
