# Supplementary material for: An LC-MS untargeted metabolomic comparison between three blood microsampling devices, whole blood, and plasma
Source: Metabolomics. 2026 May 29;22(3):89. doi: 10.1007/s11306-026-02424-6 (PMC13221320; doi:10.1007/s11306-026-02424-6)
Supplement: Supplementary file 1 [file 11306_2026_2424_MOESM2_ESM.pdf]

# LCMS QC Reference Material

CONTENTS

I. INTRODUCTION

II. STORAGE & STABILITY

III. EXAMPLES OF USING THE LCMS QCRM

IV. ORDERING INFORMATION

## I. INTRODUCTION

The Quality Control Reference Materials (QCRM) portfolio is a unique collection of standards and mixtures. These products allow the user to evaluate and benchmark their chromatography system before analysis of critical material. The products in the portfolio are all specially formulated based on the expertise of Waters scientist.

The LCMS QCRM is a 9 component mix used to provide a comprehensive reference standard for use with LC/MS or MS instrumentation with a wide variety of conditions and methods.

- The compounds in this mix give a mixture of responses in ESi (+-) and APCi+
- Covers a wide range of m/z
- Optimized concentration to provide a more equal response by component in ESI+ mode
- Provides a separation in a range of chromatographic conditions used to benchmark instrument performance

**Table 1. Individual Components in the LCMS QCRM Mix**

| Component | Empirical Formula | Exact Mass (as [M+H]+) | Exact Mass (as [M+H]–) | Concentration for analysis (µg/mL) |
|---|---|---|---|---|
| Acetaminophen | $C_8H_9NO_2$ | 152.0712 | 150.0555 | 10 |
| Caffeine | $C_8H_{10}N_4O_2$ | 195.0882 | | 1.5 |
| Sulfaguanidine | $C_7H_{10}N_4O_2S$ | 215.0603 | 213.0446 | 5 |
| Sulfadimethoxine | $C_{12}H_{14}N_4O_4S$ | 311.0814 | 309.0658 | 1 |
| Val-Tyr-Val | $C_{19}H_{29}N_3O_5$ | 380.2185 | 378.2029 | 2.5 |
| Verapamil | $C_{27}H_{38}N_2O_4$ | 455.2910 | | 0.2 |
| Terfenadine | $C_{32}H_{41}NO_2$ | 472.3216 | | 0.2 |
| Leucine-Enkephalin | $C_{28}H_{37}N_5O_7$ | 556.2771 | 554.2615 | 2.5 |
| Reserpine | $C_{33}H_{40}N_2O_9$ | 609.2812 | | 0.6 |

## II. STORAGE AND STABILITY

The standard mix comes in a Waters Crimp Top vial for direct injection and contains 500 µL total in 1:10 in ACN:LC/MS Grade Water.

The standard mix is shipped at ambient temperatures. It is highly recommended that upon receipt, the standard should be refrigerated at 4 °C for short term and for long term stored frozen.

## III. EXAMPLES OF USING THE LCMS QCRM

In this section, two example methods are discussed to allow the user to get an idea and understanding of how this standard can be used under different conditions. The user is not aiming for an exact replicate of these LC/MS chromatograms, however this is provided as guidance. The advantage of this standard is that it can be used with any column under different LC conditions. Therefore, based on the information shown below, the user can customize this standard to their specific method.

Shown in Figure 2 is a representative MS Spectrum under the following conditions listed in Table 2.:

**Table 2: Gradient Table for Figure 1**

| Time/min | Flow rate/ mL min | %A | %B | Curve |
|---|---|---|---|---|
| 0.0 | 0.5 | 100 | 0 | – |
| 3.5 | 0.5 | 0 | 100 | 6 |
| 4.5 | 0.5 | 100 | 0 | 11 |
| Total analysis time = 5 min | | | | |

Column: ACQUITY® HSS T3, 2.1 x 100 mm, 1.8 µm
Mobile phase A: Water + 0.1 % formic acid
Mobile phase B: Acetonitrile
Injection volume: 1 µL

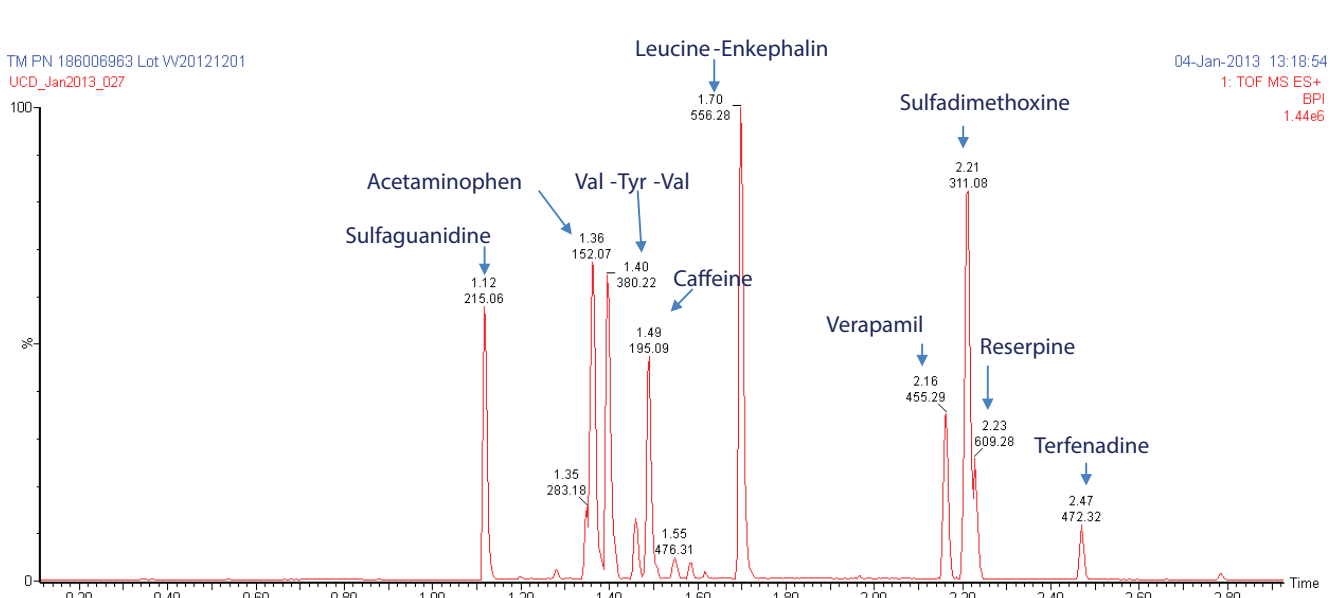


*Figure 1: TOF MS ES+ Spectrum of the LCMS QCRM Standard using ACN Mobile Phase with a SYNAPT® G2-S MS operated in positive ion mode in high resolution mode*

**Table 3: Gradient Table for Figure 2**

| Time/min | Flow rate/ mL min | %A | %B | Curve |
|---|---|---|---|---|
| 0.0 | 0.5 | 100 | 0 | – |
| 3.5 | 0.5 | 0 | 100 | 6 |
| 4.5 | 0.5 | 100 | 0 | 11 |
| Total analysis time = 5 min | | | | |

Column: ACQUITY HSS T3, 2.1 x 100 mm, 1.8 µm
Mobile phase A: Water + 0.1 % Formic acid
Mobile phase B: Methanol + 0.1 % Formic acid
Injection volume: 1 µL

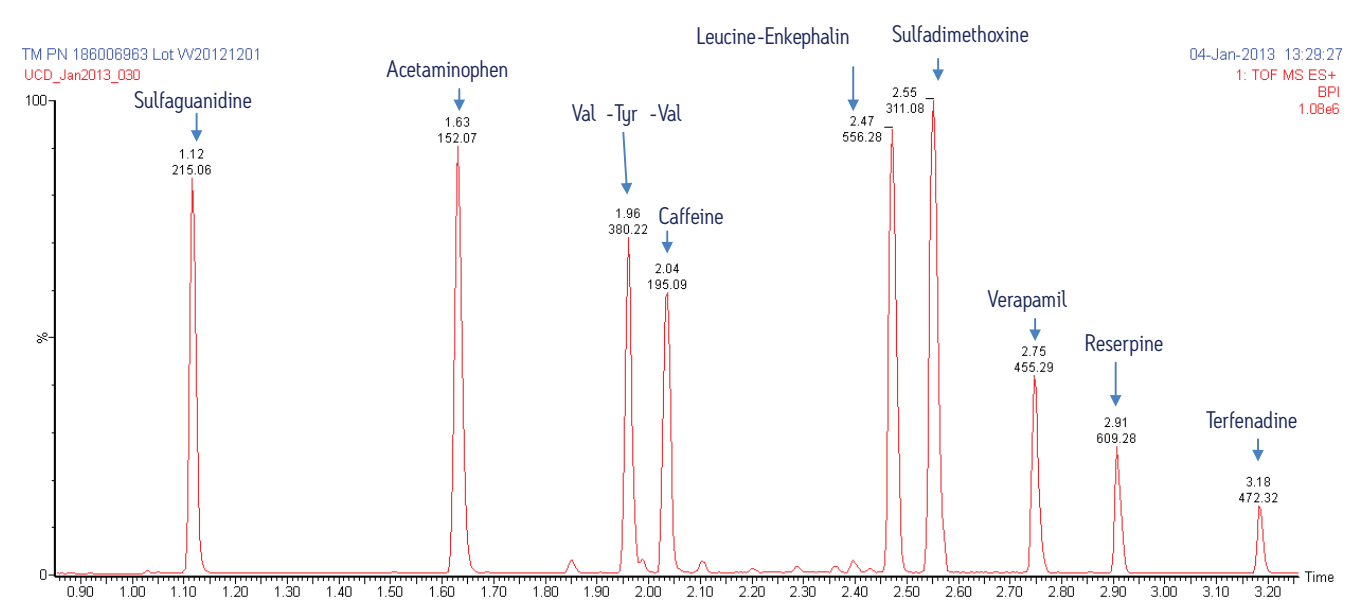


*Figure 2: TOF MS ES+ Spectrum of the LCMS QCRM Standard using Methanol Mobile Phase with a SYNAPT G2-S MS operated in positive ion mode in high resolution mode*

## IV. ORDERING INFORMATION

| Description | Part Number |
|---|---|
| LCMS QC Reference Standard | 186006963 |
| Neutrals QCRM | 186006360 |
| Reversed-Phase QCRM | 186006363 |
| Preparative Chromatography Mix Standard | 186006703 |
| HILIC QCRM | 186007226 |
| QDa QCRM | 186007345 |
| Quad LCMS QCRM | 186007362 |
| UPC$^2$ QCRM | 186007950 |
| AutoPurification System Standard | 716000765 |

Waters
THE SCIENCE OF WHAT'S POSSIBLE.®

**Waters Corporation**
34 Maple Street
Milford, MA 01757 U.S.A.
T: 1 508 478 2000
F: 1 508 872 1990
www.waters.com

Waters, The Science of What's Possible, ACQUITY, and Synapt are registered trademarks of Waters Corporation. All other trademarks are the property of their respective owners.

©2014 Waters Corporation. Produced in the U.S.A. October 2014 720004615EN Rev B AW-PDF
