# Supplementary material for: An LC-MS untargeted metabolomic comparison between three blood microsampling devices, whole blood, and plasma
Source: Metabolomics. 2026 May 29;22(3):89. doi: 10.1007/s11306-026-02424-6 (PMC13221320; doi:10.1007/s11306-026-02424-6)
Supplement: Supplementary file 2 [file 11306_2026_2424_MOESM1_ESM.docx]

**Supplementary Material 1: Methods**

**Optimization of sample preparation**

Before optimizing the analytical method, we conducted a literature review to identify preferred conditions for BµS untargeted metabolomics (Couacault et al., 2024). Some authors have already investigated the effect of the composition of the extraction mixture (Ward et al., 2021). However, as shown in the table below, studies in BµS metabolomics have reported different agitation conditions without evaluating their effects on the extracted metabolome. Therefore, we considered this research gap of interest.

Table 1 Summary of agitation conditions used in previous BµS untargeted metabolomics studies

| Entry | Reference | Extraction method | Speed (rpm) | Agitation time (min) |
| --- | --- | --- | --- | --- |
| 1 | (Sebaa et al., 2023) | Agitation | Not specified | 120 |
| 2 | (Alodaib et al., 2023) | Thermomixer | 600 | 120 |
| 3 | (Guo et al., 2023) | Shaking | 750 | 30 |
| 4 | (Ottosson et al., 2023) | Shaking  Shaking | 450  600 | 45  15 |
| 5 | (Taibl et al., 2023) | Vortex  Ultrasound | 5000  Not applicable | 10  20 |
| 6 | (Schneider et al., 2023) | Vortex  Ultrasound  Vortex  Shaking  Ultrasound | Not specified  Not applicable  Not specified  900  Not applicable | Not specified  15  Not specified  20  15 |
| 7 | (Mak et al., 2023) | Vortex | Not specified | 60 |
| 8 | (Volani et al., 2023) | Ultrasound  Vortex | Not applicable  1200 | 15  60 |

Besides vortexing and centrifugation to precipitate proteins, we decided to compare three agitation conditions: ultrasound, homogenizer, and shaker. The agitation conditions were first tested on the Whatman™ device using three sample volumes (punch sizes) and a mixture of water, methanol, and acetonitrile for metabolite extraction. We proposed agitation for 2 minutes during the extraction. Table 2 and Table 3 summarize the conditions evaluated and number of replicates

Table 2 Summary of extraction conditions used during the optimization of the analytical workflow

| Sample | Whatman | | | | | | |
| --- | --- | --- | --- | --- | --- | --- | --- |
| Extraction mixture | 40 µL H2O | | | | | | |
|  | 10 s vortex | | | | | | |
|  | 160 µL MeOH/ACN (1:1 (v/v)) | | | | | | |
|  | 10 s vortex | | | | | | |
| Agitation conditions | **Option 1** | | **Option 2** | | | **Option 3** | |
|  | 2 min | | 15 min | | | 2 min | |
|  | Ultrasound | Shaker | | 520 rpm | Homogenizer | | 4°C at 4 m/s |
|  | 15 min | | 2 min | | | 15 min | |
|  | On ice | | On ice | | | On ice | |
| Centrifugation | 10 min 17,000×g 4°C | | | | | | |
| Filtration | 0.2 µm PTFE | | | | | | |

Table 3 Number of replicates evaluated during the optimization on Whatman™ cards

| Sample Size | Extraction Technique | Samples (n) | Blanks (n) | Total |
| --- | --- | --- | --- | --- |
| 3-mm punch | Ultrasound | 3 | 1 | 4 |
| 3-mm punch | Homogenizer | 3 | 1 | 4 |
| 3-mm punch | Shaker | 3 | 1 | 4 |
| 6-mm punch | Ultrasound | 3 | 1 | 4 |
| 6-mm punch | Homogenizer | 3 | 1 | 4 |
| 6-mm punch | Shaker | 3 | 1 | 4 |
| 6-mm punches (x2) | Ultrasound | 3 | 1 | 4 |
| 6-mm punches (x2) | Homogenizer | 3 | 1 | 4 |
| 6-mm punches (x2) | Shaker | 3 | 1 | 4 |

**Extraction across devices**

Shaking and the intermediate concentration were chosen for the rest of the study. The protocol was tested and proved suitable on whole blood, plasma, Mitra®, Capitainer®, and Whatman™. The volume used for all the samples was 20 µL plus 400 µL extraction mixture (80 µL of H2O, 160 µL of MeOH, and 160 µL of ACN).

Table 4 Number of replicates evaluated during the extraction across devices

| Sample type | Samples (n) | Blanks (n) | Total |
| --- | --- | --- | --- |
| Background | -- | 1 | 1 |
| whole blood | 3 | -- | 3 |
| plasma | 3 | -- | 3 |
| Mitra® | 3 | 1 | 4 |
| Capitainer® | 3 | 1 | 4 |
| Whatman™ | 3 | 1 | 4 |

**Device and Sex Based Metabolic Differentiation**

After demonstrating that the optimized extraction method was suitable for the five sample types, it was applied on the samples of 10 volunteers (Table 4) to evaluate the ability of BµS devices to represent relevant biological information, compared to conventional liquid matrices. Table 5 summarises the types of samples collected for this part of the study.

Table 5 Blood sample codes and demographic characteristics

| Code | Sex | Age (years) | Hematocrit |
| --- | --- | --- | --- |
| E01 | Male | 19 | 47.1 |
| E02 | Male | 29 | 43.9 |
| E03 | Male | 25 | 48.0 |
| E04 | Male | 21 | 48.0 |
| E05 | Female | 26 | 42.1 |
| E06 | Male | 29 | 41.1 |
| E07 | Female | 30 | 39.5 |
| E08 | Female | 30 | 43.5 |
| E09 | Female | 32 | 36.2 |
| E10 | Female | 23 | 42.5 |
| E11 | Pooled sample | | 42.8 |

Table 6 Type of samples used in the study

| **Sample type** | | **Abbr.** | **Application volume** |
| --- | --- | --- | --- |
| Blood |  | B | 20 µL |
| Plasma |  | P | 20 µL |
| Mitra |  | M | 20 µL |
| Capitainer |  | C | 2 × 10 µL |
| Whatman |  | W | 20 µL |

**LC-MS analysis**

In detail, liquid chromatography was performed on a 1290 Infinity Binary UPLC (Agilent Technologies). Reversed-phase separation was employed using a Zorbax Eclipse XDB-C18 column (2.1 × 100 mm, 1.8 μm, Agilent Technologies, Palo Alto, CA, USA) at 50°C as the stationary phase. The mobile phase consisted of ultra-pure water (eluent A) and MeOH (eluent B), both containing 0.1% v/v formic acid. The eluent flow rate was 0.4 mL/min. The elution gradient profile was as follows (t [min], %B): (0, 2), (10, 100), (14.5, 100), (14.51, 2), (16.5, 2).

HILIC phase was employed using an Acquity UPLC® BEH Amide column (2.1 × 100 mm, 1.7 μm, Waters Corporation, Milford, MA, USA) at 45°C as the stationary phase. The mobile phase consisted of 50% v/v ACN in water (eluent A) and 90% v/v ACN in water (eluent B), both containing 20 mM ammonium formate (pH 3). The eluent flow rate was 0.6 mL/min. The elution gradient profile was as follows (t [min], %B): (0,100), (2.5,100), (10, 0), (10.01, 100), (12.5, 100).

Mass spectrometry was performed on an Agilent 6546 Q-TOF with a Jet Stream ESI ion source. Full-scan MS data were acquired with a fragmentor voltage of 100 eV over the 50-1700 m/z range. For data-dependent acquisition MS/MS analysis, the scan ranges were 50–1700 m/z, 50-250 m/z, 250-750 m/z, and 600-900 m/z. The four most abundant precursor ions were automatically selected for MS/MS fragmentation, excluded after two acquired MS/MS spectra, and released again from the exclusion list after 0.25 min. The collision energies used for the MS/MS analysis were (±)10, 20, and 40 eV. Both negative and positive ionization modes were used in the study.

1 µL of each standard and 2 µL of each sample were injected for analysis. The QC mixed sample was injected before the sample to equilibrate the column, and after every 9 samples. For the MS/MS fragmentation, plasma, whole blood, and Mitra samples were used.

MassHunter Acquisition B.05.01 software (Agilent Technologies) was used to acquire raw data from the four analytical modes: RP positive (RP+), RP negative (RP-), HILIC positive (HILIC+), and HILIC negative (HILIC-). The analytical worklist or sequence was ordered as follows: 2 blanks, 2 standard mixtures, 10 QC injections for all modes, sample injections, QC injections every 9 samples, QC MS/MS injections, and blanks. The samples’ order followed the Wranglr randomization. The standard mixtures were used to verify that retention times and abundances were within the usual ranges.

Table 7 Summary of chromatographic conditions

|  | | RP | HILIC |
| --- | --- | --- | --- |
| Stationary phase | C18 | | BEH Amide |
| Eluent A | Water | | 50% (v/v) ACN in water |
| Eluent B | Methanol | | 90% (v/v) ACN in water |
| Comment | A and B contained 0.1% (v/v) formic acid | | A and B contained 20 mM ammonium formate |
| Temperature (°C) | 50 | | 45 |
| Flow (mL/min) | 0.4 | | 0.6 |

Figure 1 RP and HILIC eluents gradient

**HUMAN consortium studies comparison**

Five laboratories, members of the HUMAN doctoral Network (human-dn.eu), received Mitra®, Capitainer®, Whatman™, whole blood, and plasma to evaluate whether BµS could affect metabolomic results compared with conventional matrices. Table 7 compares our study with other studies from the same consortium that have already been published.

The studies were conducted on the same set of samples to address the same research question, but from different approaches. Some were liquid chromatography–mass spectrometry (LC-MS) analyses, while others were capillary electrophoresis–mass spectrometry (CE-MS) and gas chromatography–mass spectrometry (GC-MS) analyses. Similarly, they have optimized different analytical parameters and focused on different types of metabolites.

Our study had an untargeted metabolomics approach. Unlike the other studies, it compared different sample concentrations and different agitation conditions during the extraction. Additionally, it compared the BµS to (1) whole blood, (2) besides whole blood, compared also to plasma, and (3) evaluated the combined effect of different analytical factors: agitation conditions, sample concentrations, LC-MS modes (reverse-phase (RP) and hydrophilic-interaction chromatography (HILIC), in positive (+) and negative (-) ionization), and several tandem mass spectrometry (MS/MS) fragmentation ranges.

Table 8 HUMAN studies comparison

| Parameter/study | Our study | (Thaitumu et al., 2025) | (Londoño-Osorio, Martínez, et al., 2025) | (Londoño-Osorio, de Sá Silva, et al., 2025) | (Marques de Sá e Silva et al., 2025) |
| --- | --- | --- | --- | --- | --- |
| Target Analytes | Untargeted Metabolomics | Untargeted Metabolomics | Lipidomics | Polar Metabolites | Untargeted Metabolomics |
| Analysis | LC-MS qToF RP and  HILIC | LC-MS qToF RP | LC-MS qToF RP | CE-MS | GC-MS  Nonpolar DBS-5 |
| Ionization | +/- ionization | + ionization | +/- ionization | +/- ionization | Electron ionization |
| Mass Range | 50–1700 m/z,  50-250 m/z,  250-750 m/z,  and 600-900 m/z | 40 to 800 m/z | 100 to 1100 m/z | Not specified | 50 - 600 m/z |
| Optimization | Sample concentration Agitation conditions | Extraction mixtures composition | No | Extraction mixtures proportion | Extraction mixtures composition |
| Source for microsamples | Venous blood | Venous blood | Venous blood | Venous blood | Venous blood (Additional study including capillary samples) |
| Reference Matrix | Whole Blood and plasma | Plasma | Plasma | Plasma | Plasma  (Additional study including capillary samples and whole blood) |
| Extraction Solvent | 80 μL of H2O +  320 μL of MeOH/ACN (1:1 (v/v)) | **For samples**  20 µL H2O + 350 µL MeOH/H2O (60:40 (v/v))  **For Plasma** 350 µL ACN/MeOH (70:30 (v/v)) | **For samples**  50 µL H2O  **For samples and plasma** 175 µL MeOH +175 µL MTBE | 80 µL MeOH/H2O (1:1) | 20 µL H2O +  320 µL MeOH |
| Extraction Method | Shaker  (2 min)  (15 min on ice) | Vortex (briefly) Agitation  (5 min) Ultrasound (15 min) | **For samples** Vortex  (8 min) Ultrasound (15 min) **For plasma** Vortex (30 min) | Vortex (2 min) Ultrasound  (15 min) | Vortex and Ultrasound  (10 min) |
| Centrifuge | 17,000×g for 10 min  at 4 °C | 6720× g for 10 min  at 4 ◦C | 16,100 x g for 15 min  at 15 ◦C | 16,100 ×g  for 1 h  at 4 ◦C | 6720g for 10 min at 4 °C |
| Additional steps | No | Evaporation Reconstitution Vortex and  centrifugation | No | No | Evaporation Derivatization Vortex + Incubation  (90 min) Vortex + Incubation  (30 min) Complete derivatization  (+ 6 h) |
| Internal standards | No | Yes | Yes | Yes | Yes |

Alodaib, A. N., Nimer, R. M., Alhumaidy, R., Alhenaky, A., Abdel Jabar, M., AlMalki, R. H., & Abdel Rahman, A. M. (2023). Biomarker discovery in galactosemia: Metabolomics with UPLC/HRMS in dried blood spots. Frontiers in Molecular Biosciences, 10, 1154149. https://doi.org/10.3389/fmolb.2023.1154149

Couacault, P., Avella, D., Londoño‐Osorio, S., Lorenzo, A. S., Gradillas, A., Kärkkäinen, O., Want, E., & Witting, M. (2024). Targeted and untargeted metabolomics and lipidomics in dried blood microsampling: Recent applications and perspectives. Analytical Science Advances, 5(5–6), e2400002. https://doi.org/10.1002/ansa.202400002

Guo, X., Zhou, L., Wang, Y., Suo, F., Wang, C., Zhou, W., Gou, L., Gu, M., & Xu, G. (2023). Development of a fast and robust liquid chromatography-mass spectrometry-based metabolomics analysis method for neonatal dried blood spots. Journal of Pharmaceutical and Biomedical Analysis, 230, 115383. https://doi.org/10.1016/j.jpba.2023.115383

Londoño-Osorio, S., de Sá Silva, D. M., Martínez, S., Theodoridis, G., Gradillas, A., García, A., Barbas, C., & González-Ruiz, V. (2025). Integrating blood microsampling devices with CE-MS for untargeted analysis of polar metabolomics. Microchemical Journal, 218, 115278. https://doi.org/10.1016/j.microc.2025.115278

Londoño-Osorio, S., Martínez, S., Avgerinou, G., Petridou, A., Mougios, V., Villaseñor, A., García, A., Gradillas, A., & Barbas, C. (2025). Deep insights into microsampling technologies for global lipidomic profiling of human whole blood using high-throughput LC-MS. Microchemical Journal, 213, 113760. https://doi.org/10.1016/j.microc.2025.113760

Mak, J., Peng, G., Le, A., Gandotra, N., Enns, G. M., Scharfe, C., & Cowan, T. M. (2023). Validation of a targeted metabolomics panel for improved second-tier newborn screening. Journal of Inherited Metabolic Disease, 46(2), 194–205. https://doi.org/10.1002/jimd.12591

Marques de Sá e Silva, D., Thaitumu, M., Virgiliou, C., Tiganouria, A., Rey-Stolle, F., Avgerinou, G., Petridou, A., Mougios, V., Theodoridis, G., & Gika, H. (2025). Toward minimally invasive metabolomics: GC-MS metabolic fingerprints of dried blood microsamples in comparison to plasma. Analyst. https://doi.org/10.1039/D5AN00937E

Ottosson, F., Russo, F., Abrahamsson, A., MacSween, N., Courraud, J., Nielsen, Z. K., Hougaard, D. M., Cohen, A. S., & Ernst, M. (2023). Effects of Long-Term Storage on the Biobanked Neonatal Dried Blood Spot Metabolome. Journal of the American Society for Mass Spectrometry, 34(4), 685–694. https://doi.org/10.1021/jasms.2c00358

Schneider, T. D., Kraemer, T., & Steuer, A. E. (2023). Untargeted Metabolomics Profiling for Determination of the Time since Deposition of Biofluids in a Forensic Context: A Proof-of-Concept for Urine, Saliva, and Semen in Addition to Blood. Analytical Chemistry, acs.analchem.3c02707. https://doi.org/10.1021/acs.analchem.3c02707

Sebaa, R., AlMalki, R. H., Alseraty, W., & Abdel Rahman, A. M. (2023). A Distinctive Metabolomics Profile and Potential Biomarkers for Very Long Acylcarnitine Dehydrogenase Deficiency (VLCADD) Diagnosis in Newborns. Metabolites, 13(6), 725. https://doi.org/10.3390/metabo13060725

Taibl, K. R., Dunlop, A. L., Barr, D. B., Li, Y.-Y., Eick, S. M., Kannan, K., Ryan, P. B., Schroder, M., Rushing, B., Fennell, T., Chang, C.-J., Tan, Y., Marsit, C. J., Jones, D. P., & Liang, D. (2023). Newborn metabolomic signatures of maternal per- and polyfluoroalkyl substance exposure and reduced length of gestation. Nature Communications, 14(1), 3120. https://doi.org/10.1038/s41467-023-38710-3

Thaitumu, M. N., De Sá E Silva, D. M., Louail, P., Rainer, J., Avgerinou, G., Petridou, A., Mougios, V., Theodoridis, G., & Gika, H. (2025). LC-MS-Based Global Metabolic Profiles of Alternative Blood Specimens Collected by Microsampling. Metabolites, 15(1), 62. https://doi.org/10.3390/metabo15010062

Volani, C., Malfertheiner, C., Caprioli, G., Fjelstrup, S., Pramstaller, P. P., Rainer, J., & Paglia, G. (2023). VAMS-Based Blood Capillary Sampling for Mass Spectrometry-Based Human Metabolomics Studies. Metabolites, 13(2), 146. https://doi.org/10.3390/metabo13020146

Ward, C., Nallamshetty, S., Watrous, J. D., Acres, E., Long, T., Mathews, I. T., Sharma, S., Cheng, S., Imam, F., & Jain, M. (2021). Nontargeted mass spectrometry of dried blood spots for interrogation of the human circulating metabolome. Journal of Mass Spectrometry, 56(8), e4772. https://doi.org/10.1002/jms.4772
