# Supplementary material for: An LC-MS untargeted metabolomic comparison between three blood microsampling devices, whole blood, and plasma
Source: Metabolomics. 2026 May 29;22(3):89. doi: 10.1007/s11306-026-02424-6 (PMC13221320; doi:10.1007/s11306-026-02424-6)
Supplement: Supplementary file 3 [file 11306_2026_2424_MOESM3_ESM.docx]

**Supplementary Material 3: Quality Control (QC) information and metrics**

During the device- and sex-based metabolic differentiation (10 volunteer samples), QC samples were prepared by collecting and pooling 15 µL from all samples before filtration (0.2 µm PTFE membranes). The QC samples were used to condition the column with 10 injections at the beginning of each run. Then, they were injected every 9 samples, for a total of 8 QC injections throughout the run. The QC injections were used to monitor instrument stability and systematic intensity drift. Later, during data analysis after drift correction, they were used as quality metrics to flag molecular features with excessive intensity variation across QC injections. The non-flagged features considered as “high-quality features” were those present in more than 70% of the QC samples, in at least 50% of samples in at least one study group, and with relative standard deviation (RSD) and D-ratio below 20% and 40%, respectively. The QC was also used to assess repeatability by monitoring how abundances varied within the same day and between days across QC injections.

The RSD was determined for the 8 QC injections within a single analytical run across the four LC-MS modes, and then we analyzed how many features (high-quality and all detected features after blank filtering) had an RSD below 30%. Below, we present tables and plots that summarize the repeatability analysis among QC injections. The information used for this summary is presented in the sheets of Supplementary Material 4.

Table 1RSD evaluation within a single RP+ analysis

| ***All detected features RP+*** | | | ***High-quality features RP+*** | | |
| --- | --- | --- | --- | --- | --- |
| ***RSD (%) limit*** | ***Frequency*** | ***Cumulative %*** | ***RSD (%) limit*** | ***Frequency*** | ***Cumulative %*** |
| **15** | 3221 | 53.66 % | **15** | 1897 | 78.55 % |
| **20** | 522 | 62.35 % | **20** | 214 | 87.41 % |
| **30** | 568 | 71.81 % | **30** | 166 | 94.29 % |
| **More** | 1692 | 100.00 % | **More** | 138 | 100.00 % |

a.

\# of Features

| RSD (%) limit | Frequency |
| --- | --- |
| 15 | 3221 |
| More | 1692 |
| 30 | 568 |
| 20 | 522 |

|  | Cumulative % |
| --- | --- |
| 15 | 53.6565% |
| More | 81.8424% |
| 30 | 91.3043% |
| 20 | 100% |

b.

\# of Features

| RSD (%) limit | Frequency |
| --- | --- |
| 15 | 1897 |
| 20 | 214 |
| 30 | 166 |
| More | 138 |

|  | Cumulative % |
| --- | --- |
| 15 | 78.5507% |
| 20 | 87.412% |
| 30 | 94.2857% |
| More | 100% |

Fig. 1 RSD evaluation within a single RP+ analysis. A. all detected features, and b. High-quality features

Table 2RSD evaluation within a single RP- analysis

| ***All detected features RP-*** | | | ***High-quality Features RP-*** | | |
| --- | --- | --- | --- | --- | --- |
| ***RSD (%) limit*** | ***Frequency*** | ***Cumulative %*** | ***RSD (%) limit*** | ***Frequency*** | ***Cumulative %*** |
| **15** | 909 | 58.46 % | **15** | 535 | 87.99 % |
| **20** | 95 | 64.57 % | **20** | 35 | 93.75 % |
| **30** | 85 | 70.03 % | **30** | 24 | 97.70 % |
| **More** | 466 | 100.00 % | **More** | 14 | 100.00 % |

a.

\# of Features

| RSD (%) limit | Frequency |
| --- | --- |
| 15 | 909 |
| More | 466 |
| 20 | 95 |
| 30 | 85 |

|  | Cumulative % |
| --- | --- |
| 15 | 58.4566% |
| More | 88.4244% |
| 20 | 94.5338% |
| 30 | 100% |

b.

\# of Features

| RSD (%) limit | Frequency |
| --- | --- |
| 15 | 535 |
| 20 | 35 |
| 30 | 24 |
| More | 14 |

|  | Cumulative % |
| --- | --- |
| 15 | 87.9934% |
| 20 | 93.75% |
| 30 | 97.6974% |
| More | 100% |

Fig. 2 RSD evaluation within a single RP- analysis. A. all detected features, and b. High-quality features

Table 3RSD evaluation within a single HILIC+ analysis

| ***All detected features HILIC+*** | | | ***High-quality Features HILIC+*** | | |
| --- | --- | --- | --- | --- | --- |
| ***RSD (%) limit*** | ***Frequency*** | ***Cumulative %*** | ***RSD (%) limit*** | ***Frequency*** | ***Cumulative %*** |
| **15** | 1878 | 68.54 % | **15** | 1090 | 87.06 % |
| **20** | 222 | 76.64 % | **20** | 86 | 93.93 % |
| **30** | 212 | 84.38 % | **30** | 48 | 97.76 % |
| **More** | 428 | 100.00 % | **More** | 28 | 100.00 % |

a.

\# of Features

| RSD (%) limit | Frequency |
| --- | --- |
| 15 | 1878 |
| More | 428 |
| 20 | 222 |
| 30 | 212 |

|  | Cumulative % |
| --- | --- |
| 15 | 68.5401% |
| More | 84.1606% |
| 20 | 92.2628% |
| 30 | 100% |

b.

\# of Features

| RSD (%) limit | Frequency |
| --- | --- |
| 15 | 1090 |
| 20 | 86 |
| 30 | 48 |
| More | 28 |

|  | Cumulative % |
| --- | --- |
| 15 | 87.0607% |
| 20 | 93.9297% |
| 30 | 97.7636% |
| More | 100% |

Fig. 3 RSD evaluation within a single HILIC+ analysis. A. all detected features, and b. High-quality features

Table 4RSD evaluation within a single HILIC- analysis

| ***All detected features HILIC-*** | | | ***High-quality Features HILIC-*** | | |
| --- | --- | --- | --- | --- | --- |
| ***RSD (%) limit*** | ***Frequency*** | ***Cumulative %*** | ***RSD (%) limit*** | ***Frequency*** | ***Cumulative %*** |
| **15** | 498 | 77.57 % | **15** | 321 | 91.98 % |
| **20** | 42 | 84.11 % | **20** | 19 | 97.42 % |
| **30** | 26 | 88.16 % | **30** | 5 | 98.85 % |
| **More** | 76 | 100.00 % | **More** | 4 | 100.00 % |

a.

\# of Features

| RSD limit | Frequency |
| --- | --- |
| 15 | 498 |
| More | 76 |
| 20 | 42 |
| 30 | 26 |

|  | Cumulative % |
| --- | --- |
| 15 | 77.5701% |
| More | 89.4081% |
| 20 | 95.9502% |
| 30 | 100% |

b.

\# of Features

| RSD limit | Frequency |
| --- | --- |
| 15 | 321 |
| 20 | 19 |
| 30 | 5 |
| More | 4 |

|  | Cumulative % |
| --- | --- |
| 15 | 91.9771% |
| 20 | 97.4212% |
| 30 | 98.8539% |
| More | 100% |

Fig. 4 RSD evaluation within a single HILIC- analysis. A. all detected features, and b. High-quality features

The previous results show that across the four modes, most detected features had an RSD below 15%. Therefore, we consider that repeatability within an analysis is adequate. This is confirmed by the median RSD calculated in all modes (Table 5).

Table 5 RSD% Median for all detected features across the four modes

| **Mode** | **Median RSD%** |
| --- | --- |
| RP+ | 13% |
| RP- | 11% |
| HILIC+ | 9% |
| HILIC- | 7% |
