# Supplementary material for: An LC-MS untargeted metabolomic comparison between three blood microsampling devices, whole blood, and plasma
Source: Metabolomics. 2026 May 29;22(3):89. doi: 10.1007/s11306-026-02424-6 (PMC13221320; doi:10.1007/s11306-026-02424-6)
Supplement: Supplementary file 4 [file 11306_2026_2424_MOESM7_ESM.docx]

**Supplementary Material 7: Additional results figures**

Fig. 1 exemplifies observed trends on different metabolites. The eight metabolites in Fig. 1 were selected as representative examples spanning a range of chemical classes (e.g., amino acids, lipids) and signal intensities to illustrate the differences in relative signal intensity across the three concentrations and homogenization conditions. This figure is illustrative as the untargeted analysis is based on the full feature set.

We categorized the samples as 'Blood-derived', which were features present across all blood-based matrices; 'Paper-associated', which are features significantly higher/unique to the paper-based devices (suggesting bleed-out from the matrix); 'Analytical background' features are present in blanks. 'Proportional features' are those whose signal intensity scales linearly with the sample concentration, while 'non-proportional features' do not, indicating a volume-dependent effect on recovery."

**Fig. 1** Representative feature trends of eight metabolites across extraction techniques and sample concentrations. **(a)** Most sample features varied proportionally with concentration and were minimally affected by extraction techniques; **(b)** some features showed non-proportional trends. **(c)** Background features remained consistent across blanks and samples. **(d)** Paper-associated features in blanks increased with paper content. Sample codes are: A, ultrasound extraction at low concentration; D, homogenizer extraction at low concentration; G, shaker extraction at low concentration; B, ultrasound extraction at medium concentration; E, homogenizer extraction at medium concentration; H, shaker extraction at medium concentration; C, ultrasound extraction at high concentration; F, homogenizer extraction at high concentration; and I, shaker extraction at high concentration. This figure is illustrative and not the basis for the untargeted statistical conclusions.

Fig. 2 shows the clustering of samples by matrix type. In the hierarchical clustering model (Fig. 2a) plasma samples clustered separately, while whole blood samples aligned more closely with BµS devices, as would have been expected by the presence of red blood cells. Among devices, Mitra® (VAMS polymer-tips) was differentiated from Capitainer® and Whatman™ (paper-based DBS). Mitra® presented the highest overall metabolite abundances, while plasma, lacking red blood cells, presented the lowest (Fig. 2b). Despite the PEG interference, Capitainer® samples closely matched whole blood molecular feature abundances (Fig. 2b).

The hierarchical dendrogram was constructed using the complete set of high-quality features (16,553) that passed initial filtering and QC thresholds. The dendrogram uses Euclidean distance and Ward’s method to cluster the samples based on overall metabolomic similarity. Data was centred and scaled for unit variance before computing the distance matrix.

**Fig. 2 (a)** Hierarchical dendrogram and **(b)** sample abundance boxplots. Plasma clustered separately from cellular matrices and presented the lowest overall metabolite abundances; Mitra® presented the highest
